# Teaching for Well-Being: The Mediating Roles of Social-Emotional Competence and Academic Buoyancy

**DOI:** 10.3390/bs16050767

**Published:** 2026-05-14

**Authors:** Christopher L. Thomas, Sarah M. Sass, Staci M. Zolkoski

**Affiliations:** 1 School of Education, University of Texas at Tyler, Tyler, TX 75799, USA; szolkoski@uttyler.edu; 2 Department of Psychology and Counseling, University of Texas at Tyler, Tyler, TX 75799, USA; ssass@uttyler.edu

**Keywords:** social emotional competence, mediation analysis, university students, teaching practices

## Abstract

Teaching practices have been identified as important predictors of perceived social-emotional competence and subsequent academic, emotional, and social outcomes in K-12 learners. However, the contribution of teaching practices to adaptive social-emotional outcomes is not as well understood in higher education contexts. Thus, the primary aim of this study was to explore the relationships between teaching quality, perceived social-emotional competence, academic buoyancy, and well-being in a university sample. The final analytic sample consisted of undergraduate and graduate students (*N* = 612) who completed the Teacher Behavior Checklist, Perceived Social Emotional Competence Scale, an academic buoyancy measure, and the Well-Being Profile Short-Form. Mediation analysis results showed that perceived social-emotional competence and academic buoyancy serially mediated the relationship between perceived teaching quality and student well-being, such that higher teaching quality was associated with greater perceived social-emotional competence, which in turn predicted higher academic buoyancy and well-being. These findings highlight the role of effective teaching in helping students develop social-emotional self-beliefs that enhance their ability to manage academic challenges and experience adaptive social-emotional outcomes.

## 1. Introduction

Student well-being is positively associated with academic performance across PK-12 and higher education settings (for a recent meta-analytic review, see Kaya & Erdem, 2021). In addition, mental health issues are negatively associated with student retention and persistence (Leow et al., 2025), while social and emotional adjustment is associated with better academic performance and retention within higher education settings (e.g., Conley, 2015; Gloria & Ho, 2003). Given the central role of social and emotional adjustment in these outcomes, research emphasizes the importance of enhancing students’ social-emotional competence (SEC) in school. Students with higher levels of SEC are more likely to remain resilient despite difficulties that may come their way (e.g., Eriksen & Bru, 2023).

Most of the literature investigating relationships between the development of SEC in school settings has focused on social emotional skills or outcomes and has overlooked motivational aspects of adaptive social and emotional regulation (for a review of this novel theoretical and conceptual framework, see Collie, 2020). In addition, much research has emphasized students’ behavioral outcomes, while comparatively little research has examined student well-being (Collie et al., 2024). It is critical to address these gaps in research, particularly given the decline in adolescent and young adult well-being worldwide (Blanchflower et al., 2025; Elharake et al., 2023). The decline in the well-being of adolescents and young adults has implications for later-life outcomes (e.g., living conditions, potential income earnings, physical health, mental health; Smith & Wesselbaum, 2024). Moreover, mixed findings in well-being interventions (Collie et al., 2024) suggest the need to identify additional factors that may support students’ well-being. Collie’s Social and Emotional Competence (SECSM) School Model addresses this limitation, and studies in K-12 environments have supported the validity of the framework. However, the SECSM has not yet been tested in higher education. The present study examines associations among perceived teaching quality, perceived SEC, academic buoyancy, and psychological well-being in a university sample, providing an initial test of the SECSM in higher education.

### 1.1. Theoretical Background

The SECSM (Collie, 2020) integrates internal mechanisms of social and emotional competence (psychological need satisfaction and autonomous motivation) with external manifestations of social and emotional competence (behaviors and skills), addressing limitations of models that focus primarily on external manifestations of SEC (for a review, see Collie, 2020). Specifically, this model proposes that the development of SEC in school-aged children and adolescents involves an iterative process between need satisfaction (perceived social-emotional autonomy, perceived social-emotional competence, and perceived relatedness) and the facilitation of socially and emotionally autonomous motivation, which promotes socially and emotionally competent behaviors. In addition, socially and emotionally competent behaviors promote the need for satisfaction in an iterative process. Different environmental influences affect this process (see Collie, 2020, Figure 1). For example, social-emotional instructional support was associated with perceived competence for emotional regulation and conflict resolution in a sample of secondary students in Australia (Collie, 2022). In higher education, perceived teaching quality may represent a salient environmental influence that supports students’ social-emotional self-beliefs, and, indeed, in at least one study involving university students, teaching quality was positively associated with social and emotional skills in university students (F. Wang et al., 2025b). Social-emotional self-beliefs, in turn, have been linked directly to well-being in higher-education populations. For example, Ciarrochi and Scott (2006) conducted a longitudinal study examining emotional competence and emotional well-being in 163 university students. They found emotional competence predicted well-being. In turn, perceived SEC may foster academic buoyancy—students’ capacity to respond adaptively to everyday academic challenges—which may ultimately support student well-being. In one recent study, emotional intelligence predicted resilience, which in turn predicted academic buoyancy in university students (Liu, 2025). Academic buoyancy has been associated with lower levels of emotional instability and neuroticism in high schoolers (Martin et al., 2013) and negatively predicted anxiety in adult graduate students (Man & Liu, 2025). F. Wang et al. (2025a) examined how social-emotional learning can be facilitated in higher education. Data was collected from two different studies: study 1 included 1539 undergraduate students, and study 2 included 499 postgraduate students. Results indicated that teachers who supported their students’ needs for autonomy, competence, and relatedness had higher levels of social-emotional learning (i.e., self-awareness, self-management, social awareness, responsible decision-making, and relationship skills).

Within the SECSM, perceived SEC refers to an individual’s perceptions of their effectiveness within different social-emotional interactions (Collie, 2020, 2022). Perceived SEC is an important part of the SEC model because it has been proposed to drive social-emotional motivation, which in turn influences behavior (Collie, 2022). Perceived SEC has been considered a “global” construct but can be broken into five different components: assertiveness, tolerance, social regulation, emotion regulation, and emotional awareness (Collie, 2022). There is evidence that the global perceived SEC factor and the five specific perceived SEC factors are all potentially related to motivation and behaviors, but in different ways (Collie, 2022). Specifically, perceived global SEC was positively associated with several types of motivation and prosocial behavior and was negatively associated with problem behaviors. Perceived social regulation competence negatively predicted external (but not autonomous) motivation and problem behaviors. Perceived tolerance competence positively predicted autonomous regulation (Collie, 2022). These findings underscore that perceived SEC, either globally or its five different facets, does not uniformly predict either motivation or behavior. Consistent with the SECSM, the present study focuses on perceived SEC as a social-emotional self-belief with the potential to serve as a mediator between perceived teaching quality and student well-being.

### 1.2. Current Study

Recent work supports the importance of motivational constructs—such as perceived SEC—to adaptive emotional and social functioning in K-12 settings (Collie, 2022). Additionally, empirical investigations have demonstrated that exposure to autonomy-supportive teaching practices enhances perceived SEC in young learners (Collie et al., 2024). However, these relationships have not been fully explored within university contexts. Thus, the purpose of the current study was to examine the associations among perceived teaching quality, perceived SEC, academic buoyancy, and psychological well-being in a university sample. Specifically, we tested whether the relationship between teaching quality and student well-being was mediated in a serial fashion by perceived social-emotional competence and academic buoyancy. Drawing on the SECSM (Collie, 2020) and previous literature, we predicted that students who perceive higher teaching quality would report stronger social emotional competence (F. Wang et al., 2025b), which would, in turn, be associated with greater academic buoyancy (Liu, 2025), and, consequently, higher levels of psychological well-being (e.g., Martin et al., 2013; Man & Liu, 2025). Formally, we tested the following hypothesis:

**H1:**  *Perceived teaching quality is positively associated with psychological well-being through the serial mediation of social-emotional competence and academic buoyancy.*

The sequential ordering of the proposed model is consistent with Collie’s (2020) SECSM, which suggests that social-emotional competence is an antecedent of adaptive academic functioning, and cross-sectional and longitudinal evidence has supported associations among teaching quality, social-emotional competence, academic buoyancy, and well-being (Bostwick et al., 2022; Chou & Sun, 2026; Collie et al., 2024; Granziera et al., 2022; C.-H. Wang et al., 2025; F. Wang et al., 2025b).

## 2. Materials and Methods

### 2.1. Participants

The initial sample included 722 undergraduate and graduate students recruited from a public university in the Southern United States. Six hundred and ten were women, 88 were men, two were transgender, and three reported other gender identities. Self-reported ethnicities included White (*n* = 422), Hispanic, Latino, or Spanish origin (*n* = 141), Black or African American (*n* = 62), Asian or Asian American (*n* = 12), American Indian or Alaska Native (*n* = 1), multi-racial (*n* = 61), and other ethnicities (*n* = 1). The mean age of the initial sample was 23.80 years (SD = 8.05). A small portion of the sample did not provide information on gender, ethnicity, or age.

Following the recommendation of Meade and Craig (2012), we included a self-report item to identify potential careless survey responses (i.e., “In your honest opinion, should we use your data?”). Participants were assured that their responses would not affect the course or extra credit awarded for participation and would be used solely to ensure that only high-quality data were included in the study. Prior work has shown that responses to this item are strongly correlated with responses to “bogus items” with an obvious correct answer, suggesting this simple self-report process is an effective way to identify potential careless responding in survey research (Meade & Craig, 2012), 90 participants were removed because they indicated their data should not be used. An additional 20 were removed due to a missing response to the item. This resulted in an analysis file consisting of 612 participants. Among those in the final analytic sample, 532 identified as women, 75 identified as men, two identified as transgender, and three reported alternative gender identities. The self-reported ethnicities of the final sample included White (*n* = 374), Hispanic, Latino, or Spanish origin (*n* = 124), Black or African American (*n* = 54), Asian or Asian American (*n* = 8), American Indian or Alaska Native (*n* = 1), multi-racial (*n* = 50), and other ethnicities (*n* = 1). The mean age of the final analytic sample was 24.04 years (*SD* = 8.27). A small portion of the sample did not provide information on gender, ethnicity, or age.

A series of independent samples *t*-tests was conducted to determine whether excluded participants significantly differed in their levels of perceived social-emotional competence, academic buoyancy, perceived teaching quality, and well-being from the final analytic sample. The results indicated that the final analytic sample reported significantly higher levels of perceived social-emotional competence, perceived teaching quality, and well-being than excluded participants. Members of the final analytic sample and excluded participants did not differ in their ratings of academic buoyancy. However, we express caution in interpreting these results as evidence of systematic difference, given the potential for careless responding among excluded participants. Independent samples *t*-test results, descriptive statistics, and effect size indices are presented in Table 1.

### 2.2. Measures

#### 2.2.1. Perceived Social Emotional Competence

We assessed students’ perceived social-emotional competence using the Perceived Social Emotional Competence Scale (PSEC; Collie, 2021). This instrument assesses 5 key domains of social emotional competence including assertiveness (i.e., “I feel capable to make sure my ideas are heard in group work at school”), tolerance (i.e., “I can listen respectfully to people who have different opinions to me”), social regulation (i.e., “I am confident I can think of the consequences before I interact with other students”), emotion regulation (i.e., “I feel capable at changing how I’m thinking if I want to feel happier about something”), and emotional awareness (i.e., “When I’m upset, I feel capable to identify the emotion I’m feeling”). Additionally, responses can be used to create a global index of perceived social-emotional competence. Participants responded to PSEC items using a 1 (Strongly Disagree) to 7 (Strongly Agree) Likert-type scale. Prior studies have provided evidence for the factorial validity, measurement invariance, and internal consistency of the PSEC in samples of secondary school learners (Collie, 2022). 

#### 2.2.2. Academic Buoyancy

We assessed academic buoyancy using a brief self-report instrument developed by Martin and Marsh (2008). Sample items include “I think I’m good at dealing with schoolwork pressures” and “I’m good at dealing with setbacks (e.g., bad mark, negative feedback on my work).” Participants responded to the buoyancy items using a 1 (Strongly Disagree) to 5 (Strongly Agree) Likert-type scale. Past studies have validated the academic buoyancy scale’s factor structure in adolescent (Martin & Marsh, 2008) and university (Thomas & Ozer, 2024) samples. Further, investigations have shown academic buoyancy scores are positively associated with conceptually related constructs (e.g., self-efficacy, academic persistence; Martin & Marsh, 2006).

#### 2.2.3. Teaching Quality

We assessed teaching quality using a modified version of the Teacher Behavior Checklist (Buskist et al., 2002). The instrument assesses caring and supportive behaviors (e.g., “polite to students,” and “accepts legitimate excuses for missing class”) as well as elements of professional competency and communication skills (e.g., “establishes clear classroom rules,” “uses clear and understandable examples”) that are associated with high-quality teaching. Additionally, instrument items can be used to create an overall index of teaching effectiveness. The original version of the instrument allows students to rate the extent to which specific instructors possess—or engage in—the identified characteristics and behaviors (e.g., “Dr. _____ frequently exhibits these behaviors”). However, we modified the response options by replacing this instructor-specific wording with “my professors” (e.g., “My professors frequently exhibit these behaviors”) to capture students’ overall perception of teaching quality. Participants responded to the teaching quality items using a 1 (My professors never exhibit this behavior) to 5 (My professors always exhibit this behavior) scale. Prior psychometric investigations have provided evidence supporting the multidimensional latent structure, test–retest reliability, and convergent validity of the original version of the instrument (Keeley et al., 2010, 2006).

#### 2.2.4. Well-Being

We assessed students’ well-being using the Well-Being Profile Short Form Instrument (5 items; Marsh et al., 2020). Guided by a multi-dimensional conceptual model, this short-form instrument contains items aligned with numerous facets of well-being including emotional stability (i.e., “I do not get easily upset”), positive relationships (i.e., “There are people with whom I can discuss intimate and personal matters”), prosocial behavior (i.e., “If a person needs help, I would do almost anything to assist”), and self-esteem (i.e., “I feel that I have a number of good qualities). Participants completed the instrument using a 1 (Completely disagree) to 9 (Completely agree) Likert-type scale. Scores on the 5-item WB-Pro in previous psychometric investigations demonstrated strong criterion-related validity evidence through strong positive correlations with the full 48-item instrument and consistent positive associations with established well-being measures (Marsh et al., 2020).

### 2.3. Procedure

All participants were recruited from research participation pools within the School of Education and the Department of Psychology and Counseling. Participation was incentivized with partial course credit or extra credit in courses that included a research pool requirement. Students who chose not to participate in this study had the opportunity to earn course credits by participating in other studies listed on the departmental SONA sites or completing alternative assignments requiring comparable time and effort. Students completed all materials using the Qualtrics survey management platform, and informed consent was obtained prior to data collection. The study was approved by the university’s institutional review board (IRB).

### 2.4. Analytic Strategy

We first investigated the structural validity of each self-report measure when applied to our undergraduate and graduate student sample. Specifically, confirmatory factor analysis using the WLSMV estimator was used to verify the latent structures of the PSEC (bi-factor model with five first-order factors; Collie, 2022), academic buoyancy (one-factor model; Martin & Marsh, 2008), teaching behavior checklist (one-factor or two-factor model; Keeley et al., 2006), and well-being (one-factor; Marsh et al., 2020) described in previous empirical reports. Model fit was assessed using comparative fit index (CFI; Bentler, 1990), Tucker–Lewis Index (TLI; Tucker & Lewis, 1973), and standardized root mean square residual (SRMR) values. A good-fitting confirmatory factor analysis model was indicated by CFI and TLI ≥ 0.90, and SRMR ≤ 0.08 (Kline, 2016). We did not consider the root mean square error of approximation (RMSEA; Steiger, 1990) because of prior work suggesting that these values can be potentially misleading in models with small degrees of freedom and when analyzing ordinal data using WLSMV (Kenny et al., 2015; Shi et al., 2020). The confirmatory factor analysis models were estimated using Mplus (Version 8.11). Next, Cronbach’s alpha values, descriptive statistics, and bivariate correlations were calculated to evaluate internal consistency and general trends in the collected data. Finally, a serial mediation model was used to test whether perceived social-emotional competence and academic buoyancy sequentially mediate the relationship between teaching quality and student well-being. Observed composite scores were created by averaging item responses for each construct and used as indicators in the mediation model. Confidence intervals were estimated using the percentile bootstrap method (*n* = 1000). Reliability analyses, descriptive statistics, bivariate correlations, and the mediation analysis were conducted using JASP (version 0.95.2; JASP Team, 2025).

## 3. Results

### 3.1. Confirmatory Factor Analysis

Model fit indices indicated that the proposed latent structures for the PSEC, academic buoyancy, and teaching quality instruments provided an adequate fit to the observed data. CFI and SRMR values suggested an adequate fit for the well-being instrument. However, the TLI value fell below the threshold, suggesting potential model misspecification. Therefore, we examined residual correlations for evidence of local misfit. All residual correlations fell below |0.20|, providing no evidence of local misfit (Higgins et al., 2026). On this basis, the well-being instrument was determined to demonstrate adequate model fit. Model fit values are presented in Table 2. Factor loadings and reliability estimates for all instruments are presented in Supplementary Tables S1–S4.

### 3.2. Internal Consistency Estimates, Descriptive Statistics, and Bivariate Correlations

Reliability analyses indicated that the primary study measures demonstrated acceptable internal consistency (a > 60; What Works Clearinghouse, 2022). Although Cronbach’s alpha values were acceptable according to What Works Clearinghouse guidelines, it is important to note that the internal consistency estimate for our well-being measure falls just above the 0.60 threshold. Given the conceptual importance of well-being to our investigation, we decided to estimate mean inter-item correlation values to provide further evidence of reliability for the measurement tool. Prior work has suggested that mean inter-item correlation values may provide a more accurate estimate of internal consistency when using brief measurement tools. The inter-item correlation value was interpreted using the guidelines outlined by Clark and Watson (1995) and Streiner (2003), with values between 0.15 and 0.50 indicating acceptable internal consistency. Our results indicated that our measure of student well-being demonstrated acceptable internal consistency in the current study (Mean inter-item correlation = 0.22). Correlation results showed small to moderate positive correlations among teaching quality, perceived social-emotional competence, academic buoyancy, and well-being estimates (*rs* = 0.20–0.59). The Cronbach’s alpha values, correlation coefficients, and descriptive statistics are presented in Table 3.

### 3.3. Mediation Analysis

The variables included in the serial mediation model accounted for a modest amount of variance in social-emotional competence (R2 = 0.14), academic buoyancy (R2 = 0.19), and well-being (R2 = 0.38).

#### 3.3.1. Direct Effects

The mediation analysis results showed that perceived teaching quality was positively and significantly associated with perceived social-emotional competence (*β* = 0.38, *p* < 0.001) and well-being (*β* = 0.09, *p* = 0.004). Perceived social-emotional competence was positively and significantly associated with academic buoyancy (*β* = 0.42, *p* < 0.001) and student well-being (*β* = 0.48, *p* < 0.001). The results also revealed a statistically significant positive relationship between academic buoyancy and well-being (*β* = 0.16, *p* < 0.001). Interestingly, the direct relationship between perceived teaching quality and academic buoyancy was non-significant (*β* = 0.03, *p* = 0.360).

#### 3.3.2. Indirect Effects

A review of simple indirect effects showed that the relationship between perceived teaching quality and well-being was mediated by perceived social-emotional competence (*β* = 0.18, *p* < 0.001). The simple indirect effect involving perceived teaching quality, academic buoyancy, and well-being was non-significant (*β* = 0.00, *p* = 0.360). Most importantly, our results indicated that social-emotional competence and academic buoyancy serially mediate the relationship between overall teaching quality and learner well-being. These findings suggest that high-quality teaching enhanced students’ social-emotional competence. Social-emotional competence subsequently strengthened their academic buoyancy and, in turn, their overall well-being. Indirect effect estimates are presented in Table 4.

#### 3.3.3. Sensitivity Analyses

Given the cross-sectional nature of the data, we examined several alternative model orderings to assess the robustness of the proposed serial mediation structure. The first competing model reversed the order of the mediating variables, specifying that academic buoyancy followed by perceived social-emotional competence as mediators of the teaching quality–well-being relationship. Results indicated that the sequential indirect effect under this alternative ordering was statistically significant (*β* = 0.03, *p* < 0.001). The second competing model was specified such that well-being predicted perceived teaching quality, with social-emotional competence and academic buoyancy serving as mediating variables. Results indicated that the sequential indirect effect under this reversed specification was non-significant (*β* = 0.003, *p* = 0.715). These sensitivity analyses provide partial support for the robustness of the proposed sequential mediation model. We elected to retain the proposed sequential ordering due to its alignment with the SECSM (Collie, 2020) and prior cross-sectional and longitudinal evidence supporting the proposed model ordering (Bostwick et al., 2022; Chou & Sun, 2026; Collie et al., 2024; F. Wang et al., 2025b).

## 4. Discussion

The primary purpose of the study was to gain a better understanding of the relationships among teaching quality, perceived social-emotional competence, academic buoyancy, and well-being in a university sample. One key finding was that students’ perceived social-emotional competence was strongly related to academic buoyancy and well-being. This finding is consistent with work in university samples demonstrating that students with high emotional self-efficacy (e.g., trait emotional intelligence) are better able to manage challenging situations (Di Fabio & Saklofske, 2018; Thomas & Zolkoski, 2020) and are more likely to demonstrate behaviors and dispositions that are characteristic of strong subjective well-being such as prosocial behaviors (H. Wang et al., 2021), positive social relationships (Schutte et al., 2001), and elevated self-esteem (Schutte et al., 2002) compared to their low emotional self-efficacy peers. Additionally, these results are consistent with work guided by the Social and Emotional Competence School Model, which has shown that perceived social-emotional competence supports coping potential and emotional well-being in adolescent learners (Collie, 2022; Collie et al., 2024). As noted in the literature, the benefits of perceived social-emotional competence likely follow from the broad energizing effects of ability judgments. Specifically, students who are confident in their emotional and social abilities are better able to organize and implement skills (e.g., behavioral, cognitive, social) directed at goal attainment within and beyond traditional academic settings (Collie & Martin, 2024).

Most importantly, and in support of our primary hypothesis, our results demonstrated that the association between students’ overall perception of teaching quality and well-being is mediated in a serial manner by perceived social-emotional competence and academic buoyancy. These findings are consistent with past work within K-12 settings demonstrating that contextual resources—such as teaching practices that support students’ autonomy through interest development, provide structure via clear expectations and effective goal-setting, and foster relatedness through a supportive classroom atmosphere—enhance students’ perceived ability to appraise and regulate emotions and navigate academically oriented social situations by satisfying fundamental psychological needs underlying the development of adaptive self-beliefs (Collie, 2022; Collie & Martin, 2024). These social-emotional self-beliefs in turn bolster learners’ ability to manage academic challenges and maintain emotional and social well-being (Collie et al., 2024).

### 4.1. Theoretical Implications

Our findings contribute to the literature in several ways. First, our findings provide initial evidence supporting the viability of the SECSM in explaining the development and outcomes of social-emotional competence within university contexts. Specifically, the current investigation suggests that teaching quality may be linked to undergraduate and graduate students’ well-being because it supports perceived social-emotional competence, which in turn promotes academic buoyancy. Second, our investigation provides preliminary validity and reliability evidence of the Social Emotional Competence Scale (Collie, 2021) within university student samples. This preliminary psychometric evidence represents a meaningful first step in building measurement infrastructure to support investigations of the motivational aspects of social-emotional functioning in post-secondary contexts.

### 4.2. Practical Implications

Previous research has focused on learning environments and students’ social-emotional skills in primary and secondary students (e.g., Mahoney et al., 2021). However, F. Wang et al. (2025a) extended these same findings in undergraduate and postgraduate students. The link between students’ perception of their teachers’ support impacts students’ social-emotional skills. Wang and colleagues’ study highlights the importance of teachers’ support for students at all levels of their academic journey. The present results suggest that the quality of teaching in higher education matters in promoting the perceived social-emotional competence of emerging adults and adult learners, which, in turn, matters in promoting academic buoyancy and overall student well-being. The present results replicate and extend similar findings of a connection between teaching practices and perceived SEC in younger students to older university-aged students. These findings have practical implications for faculty and other instructors within higher education. First, focusing on high-quality teaching practices has the potential to impact the whole student. That is, high-quality teaching practices can support a student not just academically but also in their social-emotional growth and well-being, functioning as catalysts for the development of broader competencies that extend to life and career challenges beyond academic performance (Mahmood et al., 2025). Indeed, the declining mental health of students in higher education has been the focus of attention for several decades (e.g., Auerbach et al., 2016; Blanco et al., 2008; Salimi et al., 2023; Sass et al., 2019; Sheldon et al., 2021). F. Wang et al. (2025a) found that students who perceived teachers as supportive were more likely to exhibit greater social-emotional skills. When students feel valued, supported, and respected by their teachers, they are more satisfied with the learning process and have increased social-emotional skills. Even in higher education, educators need to create an environment where students feel cared for. Present results suggest that high-quality teaching practices were associated with students’ self-reported perceived SEC, which, in turn, was associated with their well-being and ability to stick with their academic goals despite setbacks. Teachers can support the needs of students by making minor adjustments to instruction, such as providing clear expectations and feedback, and explaining the rationale behind decisions made. Additionally, it is important to build positive relationships with students. For example, instructors can solicit student feedback and consider students’ perspectives on what effective teaching and instructional support look like.

### 4.3. Limitations

The primary limitation of this study is the use of cross-sectional data, which limits our ability to make conclusions regarding the causal relationships between the variables included in the serial mediation model. Thus, we encourage future work using longitudinal or experimental designs to provide additional evidence of the mediation pathways tested in this investigation. Another limitation is related to the recruitment of students from a single university through departmental research pools. It is possible for students who elected to participate in this investigation to differ in meaningful ways from the broader university student population. Extending from this limitation, our study consisted primarily of female students pursuing degrees in fields related to education, psychology, and counseling. As such, it is unclear how well the findings will generalize to contexts with increased gender diversity or academic disciplines. We recommend recruiting university participants from many different departments and disciplines in future research. Finally, this study relied on students’ perceptions of teaching quality as assessed by a modified version of the Teacher Behavior Checklist. Although CFA results supported the factorial stability of the adapted instrument, it is possible that reframing items from ratings of a specific instructor to students’ generalized perceptions of “my professors” shifts the construct toward a broader institutional judgment, and future validation work is warranted. Future research could assess course- and instructor-level teaching quality to tease out the effects of a single professor’s teaching quality compared to a broader aggregate of teaching quality within a discipline or program. More broadly, given research documenting potential biases in student evaluations of teaching (Adams et al., 2022; Chávez & Mitchell, 2020), ratings of teaching quality may be influenced by factors other than teaching practices. Future research could address this limitation through observational methodologies of teaching practices and analysis of course and student artifacts to more comprehensively assess high-impact teaching practices.

## 5. Conclusions

Theoretical and empirical work emphasizes the importance of high-impact teaching practices in supporting social-emotional competence and adaptive academic and social outcomes in K-12 learners. Currently, investigations demonstrating associations among teaching practices, social-emotional competence, and key academic outcomes within university populations are relatively scarce. The current study attempted to address this gap in the literature by using mediation analysis to investigate the influence of teaching quality on students’ perceived social-emotional competence, academic buoyancy, and well-being. The findings showed that teaching quality was associated with student well-being through a sequential pathway. Specifically, exposure to perceived high-quality teaching was associated with stronger social-emotional self-beliefs, which in turn were associated with greater academic buoyancy. These findings highlight the potential importance of engaging, organized, and supportive classroom environments in fostering the psychological needs associated with effective social and emotional functioning in university students.

## Figures and Tables

**Table 1:** Summary of independent *t*-test results.

Outcome Variable	T-Statistic (df)	*p*-Value	Cohen’s D	Final Sample Mean (SD)	Excluded Mean (SD)
PSEC	−4.46 (704)	<0.001	−0.49	5.64 (0.85)	5.19 (1.14)
Academic Buoyancy	−1.05 (703)	0.291	−0.11	3.20 (0.90)	3.09 (1.02)
Perceived Teaching Quality	−2.68 (704)	0.007	−0.29	4.33 (0.61)	4.14 (0.79)
Well-Being	−3.08 (700)	0.002	−0.34	6.39 (1.02)	6.03 (1.06)

Note: PSEC = Perceived Social Emotional Competence.

**Table 2:** Summary of fit statistics for CFA models.

Model	CFI	TLI	SRMR
SEC Bi-Factor	0.95	0.94	0.069
TBC—Two Factor	0.96	0.95	0.036
TBC—One Factor	0.96	0.96	0.035
Academic Buoyancy	0.98	0.96	0.020
Well-Being	0.91	0.82	0.037

Note. CFI = Comparative Fit Index; TLI = Tucker–Lewis Index; SRMR = Standardized Root Mean Square Residual.

**Table 3:** Descriptive statistics, Cronbach’s alpha values, and bivariate correlations among the variables of interest.

Variable	M	SD	a	1	2	3	4
1. Social Emotional Competence	5.64	0.85	0.93	-			
2. Teaching Quality	4.33	0.61	0.97	0.38 *	-		
3. Academic Buoyancy	3.20	0.90	0.82	0.44 *	0.20 *	-	
4. Well-Being	6.39	1.02	0.64	0.59 *	0.32 *	0.40 *	-

Note: * *p* < 0.05.

**Table 4:** Serial Mediation Model Indirect Estimates.

					95% Confidence Interval		
Effect	Estimate	Std. Error	z-Value	*p*	Lower	Upper	Std. Estimate
Teaching Quality ⟶ Social Emotional Competence ⟶ Well-Being	0.31	0.039	8.07	<0.001	0.207	0.437	0.18
Teaching Quality ⟶ Academic Buoyancy ⟶ Well-Being	0.01	0.011	0.915	0.360	−0.011	0.037	0.00
Teaching Quality ⟶ Social Emotional Competence ⟶ Academic Buoyancy ⟶ Well-Being	0.04	0.012	4.10	<0.001	0.024	0.074	0.02

## Data Availability

The datasets presented in this article are not readily available due to restrictions imposed by the Institutional Review Board at The University of Texas at Tyler.

## Supplementary Materials

The following supporting information can be downloaded at: https://www.mdpi.com/article/10.3390/bs16050767/s1.
