# Supplementary material for: Teaching for Well-Being: The Mediating Roles of Social-Emotional Competence and Academic Buoyancy
Source: Behav Sci (Basel). 2026 May 14;16(5):767. doi: 10.3390/bs16050767 (PMC13203393; doi:10.3390/bs16050767)
Supplement: Supplementary file 1 [file behavsci-16-00767-s001.zip › behavsci-4177858-supplementary.pdf]

Table S1. Perceived Social Emotional Competence Scale: Factor Loadings from Bifactor Confirmatory Factor Analysis.

Items	PSEC	Assertiveness	Tolerance	Social Regulation	Emotion Regulation	Emotional Awareness
Item 1	.56	.62				
Item 2	.57	.42				
Item 3	.51	.46				
Item 4	.57	.49				
Item 5	.74		.40			
Item 6	.77		.60			
Item 7	.81		.50			
Item 8	.76		.48			
Item 9	.76			.30		
Item 10	.79			.41		
Item 11	.81			.29		
Item 12	.84			.24		
Item 13	.64				.59	
Item 14	.69				.61	
Item 15	.67				.68	
Item 16	.64				.81	
Item 17	.73					.29
Item 18	.64					.60
Item 19	.62					.72
Item 20	.60					.66

Note. All factor loadings are STDYX standardized estimates; PSEC = Perceived Social Emotional Competence.

Table S2. Well-Being: Factor Loadings from One-Factor Confirmatory Factor Analysis.

Items	Well-Being
Item 1	.47
Item 2	.42
Item 3	.63
Item 4	.60
Item 5	.82

Note. All factor loadings are STDYX standardized estimates.

Table S3. Academic Buoyancy: Factor Loadings from One-Factor Confirmatory Factor Analysis.

Items	Well-Being
Item 1	.72
Item 2	.83
Item 3	.85
Item 4	.74

Note. All factor loadings are STDYX standardized estimates.

Table S4. Teacher Behavior Checklist: Factor Loadings from One and Two Factor Confirmatory Factor Analysis Solutions.

Items	One Factor Solution	Two Factor Solution – F1	Two Factor Solution – F2
Item 1	.73	.73	
Item 2	.80		.80
Item 3	.70		.70
Item 4	.83		.83
Item 5	.81		
Item 6	.84		.85
Item 7	.85	.85	
Item 8	.85	.85	
Item 9	.81		
Item 10	.82	.83	
Item 11	.85		.86
Item 12	.85		.86
Item 13	.82	.83	
Item 14	.84		.85
Item 15	.83		.83
Item 16	.84		
Item 17	.77		
Item 18	.84	.84	
Item 19	.87	.87	
Item 20	.81	.81	
Item 21	.84		.84
Item 22	.80	.80	
Item 23	.84	.85	
Item 24	.82		.83
Item 25	.84	.84	
Item 26	.85	.85	
Item 27	.74		.74
Item 28	.82	.83	

Note. All factor loadings are STDYX standardized estimates; The two-factor structure of the TBC, was derived from Keeley et al. (2006), who excluded four items from the two-factor solution.
